# Death of Leaves from the Fumes of Lead

**Published:** 1835

**Authors:** 


### V.—Death of leaves by the fumes of Lead.

A young sugar tree, (*acer saccharinum*,) planted along one of the side walks of this city, put forth several tufts of leaves near the top of the stem, about seven feet from the ground. When they were growing luxuriantly, the box, a large one, in which the tree was enclosed for protection, was painted with white lead, in oil. By the next day the whole of them had wilted, and have since been replaced with a new crop. This was a palpable and fatal case of saturnine paralysis in the vegetable kingdom.

